# Real-World Shelf Life of Adrenaline Auto-Injectors at Pharmacies in Denmark, Finland, Sweden, and Norway

**DOI:** 10.3390/jmahp14020026

**Published:** 2026-05-01

**Authors:** Jesper Nørregaard, Christoffer Mertz, Anne Danø, Jeppe Hæstrup Kamstrup, Mille Vang Lybech

**Affiliations:** 1 ALK-Abelló Nordic A/S, 434 37 Kungsbacka, Sweden; jesper.norregaard@alk.net; 2 Eviproseon, 4320 Lejre, Denmark; christoffer.mertz@eviproseon.dk; 3 Inspiria, 2960 Rungsted Kyst, Denmark; anne.danoe@inspiria.dk; 4 Signum Life Science, 2100 Copenhagen, Denmark; jhk@signumlifescience.com; 5 ALK-Abelló, 2970 Hørsholm, Denmark

**Keywords:** adrenaline auto-injectors, anaphylaxis treatment, real-world shelf life, expiry

## Abstract

There is a well-documented gap between the prescription of adrenaline auto-injectors (AAIs) and their real-world use during anaphylaxis. Although several aspects of AAI underuse have been investigated, the potential role of shelf life in influencing patient adherence has not been quantified. This study assessed the real-world remaining shelf life of AAIs available at pharmacies in Denmark, Finland, Sweden, and Norway, using pharmacy-level stock data and pharmacy employee-reported perceptions. Across Denmark, Finland, and Sweden, the average remaining shelf life was 9.6 months, and in Norway it was 10.5 months at the point of dispensing. In Denmark, Finland and Sweden, 100%, 91%, and 94% of employees, respectively, considered shelf life an important or very important factor when dispensing AAIs to patients. Our findings suggest that patients and caregivers filling prescriptions for AAIs frequently receive devices with limited remaining shelf life, which may necessitate multiple renewals per year. This has potential implications in terms of adherence to clinical guidelines, dependence of expired devices during emergencies, patient cost, caregiver burden, and overall societal expenditure. These results highlight an unmet need for emergency treatment options with longer shelf life to better support continuous access to life-saving medicine during anaphylaxis.

## 1. Introduction

Anaphylaxis is a rapid-onset, potentially life-threatening allergic reaction that requires immediate administration of adrenaline as first-line treatment [1,2,3]. Clinical guidelines recommend that individuals at risk of anaphylaxis carry adrenaline auto-injectors (AAIs) and use them promptly at the onset of symptoms [3]. Despite the proven efficacy and availability of AAIs, real-world evidence reveals a significant gap between prescription, carriage, and actual use during anaphylactic episodes [4,5,6,7,8,9,10,11,12,13]. Although no comprehensive analysis exists across all Nordic countries, data from Denmark indicate that 24.3% of patients first seen at the emergency department of Odense University Hospital for a suspected allergic reaction and later evaluated at the Allergy Centre did not fill their AAI prescription following the diagnostic work-up [5]. A similar trend is observed internationally, as highlighted in a US study involving 2000 adults and children who had filled at least one AAI prescription which found that only 50% carried their device at all times during the previous week [14]. In addition, a US study found that only 54% of patients seen in an emergency department for food-induced anaphylaxis filled an AAI prescription within one year [8].

Barriers to AAI use and carriage include factors such as user hesitation, needle-related risks, unfilled prescriptions, device complexity, inadequate training and portability limitations [4,7,9,10,11,12]. Even when AAIs are carried, they are not always used during anaphylactic episodes. In a UK questionnaire study, an AAI was available in 79% of episodes but used in only 38%, with lower use in children than adults [6]. Similarly, a U.S. chart review found that among children with access to an AAI, one-third did not use it during food-related allergic reactions [15].

These gaps represent an unmet need with implications for patient safety, healthcare burden, and costs [16,17]. Improving adherence to AAI carriage and ensuring timely use during anaphylaxis have therefore been identified as important targets for reducing both the clinical risk of severe outcomes and the associated economic burden [18,19,20,21,22,23,24]. More generally, sustained medication adherence remains challenging across diseases and healthcare systems, particularly with respect to maintaining ongoing access to treatment and ensuring availability at the point of need [25,26,27,28].

An often overlooked factor in this context is the shelf life of AAIs. At manufacturing, shelf life ranges from 18 and 24 months, depending on dose and brand [29,30,31,32]. AAIs are prescribed as a safety precaution in the event that anaphylaxis should occur; fortunately, most patients do not experience an anaphylactic episode during the device’s shelf life. As a result, most AAIs are disposed of unused upon expiry. Consequently, patients (or caregivers of paediatric patients) must periodically refill prescriptions to ensure access to non-expired emergency treatment. A shorter remaining shelf life at the point of dispensing may increase costs and inconvenience, potentially undermining adherence and worsening outcomes during anaphylaxis [3].

The Nordic countries, Denmark, Finland, Sweden, and Norway, constitute a relevant setting for examining real-world AAI shelf life due to their publicly funded healthcare systems and similar pharmacy structures, and in all four countries AAIs are available at retail pharmacies but require a prescription. These shared characteristics allow meaningful cross-country comparison, while differences in supply chain organisation and distribution logistics may influence the remaining AAI shelf life available to patients. As no prior cross-Nordic assessment has been conducted, a combined analysis enables evaluation of whether short remaining shelf life is a consistent regional phenomenon and provides insight into variability across countries. Despite the relevance of shelf life for patient adherence, no study to date has quantified remaining AAI shelf life at the point of dispensing in any real-world setting. As an initial step towards understanding this issue, data on the in-stock shelf life of AAIs at pharmacies in Denmark, Finland, Sweden and Norway are presented, demonstrating that remaining shelf life is substantially shorter than the original manufacturing shelf life. Additionally, insights into pharmacy employees’ interactions with patients are provided, highlighting an unmet need for emergency treatment options with longer shelf life.

## 2. Materials and Methods

### 2.1. Data Collection

This study is based on interviews with employees at retail pharmacies in Denmark, Finland, Sweden, and Norway. For all countries, except Norway, expiry dates and batch numbers were collected for all AAIs in stock, regardless of dose or brand, at the employees’ workplace pharmacies. In addition, employees in Denmark, Finland and Sweden were asked about their interactions with patients concerning purchasing patterns and perceptions of the current state of AAI shelf life. This included the four questions listed in Table A1.

Pharmacies were selected to ensure geographic representation across regions, with the final sample comprising approximately 5–10% of all retail pharmacies in each country. Selection was not based on pharmacy size, ownership structure, or local demographic characteristics. The sampling strategy was designed to descriptively capture variability in real-world dispensing practices rather than to provide a statistically representative national sample.

In Norway, data were collected through physical visits to pharmacies to identify which AAI a patient, or the caregiver of a paediatric patient, would be offered if they requested a specific AAI product.

### 2.2. Data Analysis

The data collected from interviews in Denmark, Finland, and Sweden were checked for inconsistencies. Expiry dates of AAIs in stock were converted into remaining shelf life, expressed as the number of days between the date of data collection and the expiry date. Remaining shelf life was then converted into months using a standardised 30.44 days per month to facilitate comparison across countries.

The analysis was intended to characterise real-world remaining shelf life of AAIs at the point of dispensing rather than to formally test hypotheses or conduct benchmark-based comparisons between countries or subgroups.

Descriptive statistics were used to summarise stock availability, remaining shelf life, and employee-reported perceptions. Given the non-probabilistic sampling strategy, no inferential statistical tests were appropriate.

## 3. Results

### 3.1. Shelf-Life Analysis

A total of 250 interviews were conducted between 23rd of July and 16th of October 2025 across Denmark, Finland, Sweden, and Norway. In Denmark, pharmacy assistants were the most frequent respondents, whereas in Finland and Sweden most interviewees were conducted with pharmacists (Table A2). Staff type was not recorded in Norway.

Across Denmark, Finland, and Sweden, pharmacy employees reported a total of 792 AAIs in stock at the time of data collection (Table 1). Of these, 201 (25.4%) were 150 µg devices and 591 (74.6%) were 300 µg devices, consistent with the larger proportion of the patient population requiring the higher dose [33]. Stock availability varied by country: 150 µg devices were present at 52% of all pharmacies surveyed, whereas 300 µg devices were available at 90%. The average number of AAIs in stock per pharmacy was low across all countries, ranging from 1.5 to 2.1 devices for the 150 µg dose and 2.6 to 3.7 devices for the 300 µg dose.

Remaining shelf life varied substantially across countries and doses (Table 2). The mean remaining shelf life of all devices in Denmark, Finland, and Sweden combined was 9.6 months. The 150 µg dose consistently exhibited a shorter remaining shelf life than the 300 µg dose. The average remaining shelf life for 150 µg devices was 6.2 months, compared with 10.8 months for 300 µg devices. This pattern was observed across all countries and was particularly pronounced in Finland, where the average remaining shelf life of 150 µg pens was only 4.3 months. Denmark had the shortest overall average remaining shelf life of combined doses with 6.9 months, Finland was intermediate with 8.9 months, and Sweden had the longest with 10.7 months.

The range of remaining shelf life across all three countries was wide. For the combined doses, the minimum and maximum remaining shelf life ranged from 1.1 to 21.9 months, reflecting substantial heterogeneity in the stock available to patients.

In Norway, where a sample-based approach was used to identify the remaining shelf life of AAIs, the mean remaining shelf life was 10.4 months for the combined doses across 170 AAIs.

**Figure 1:**
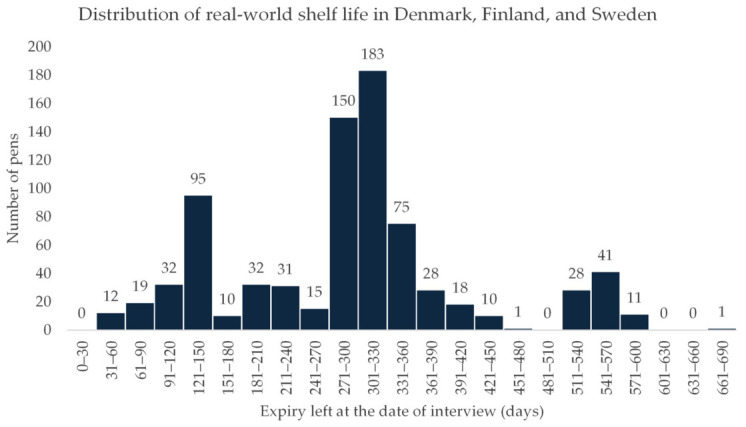
Distribution of remaining AAI shelf life of the combined 150 µg and 300 µg doses at pharmacies in Denmark, Finland, and Sweden.

Figure 1 presents the distribution of remaining shelf life for the combined doses in Denmark, Finland, and Sweden. Most AAIs, 659 of 792 (83.2%), had a remaining shelf life of 365 days or less. The most common remaining shelf-life interval was 301–330 days, corresponding to approximately 10–11 months, observed for 183 AAIs. A secondary peak of 95 AAIs with 121–150 days remaining (4–5 months) was identified, consisting predominantly of the 150 µg dose (93 of 95; not depicted in Figure 1). A minor third peak was observed at 511–600 days (17–20 months) consisting exclusively of 300 µg AAIs. Only 10.3% of AAIs identified had a remaining shelf life of more than 450 days (15 months).

### 3.2. Pharmacy Employee Shelf-Life Perception Analysis

**Figure 2:**
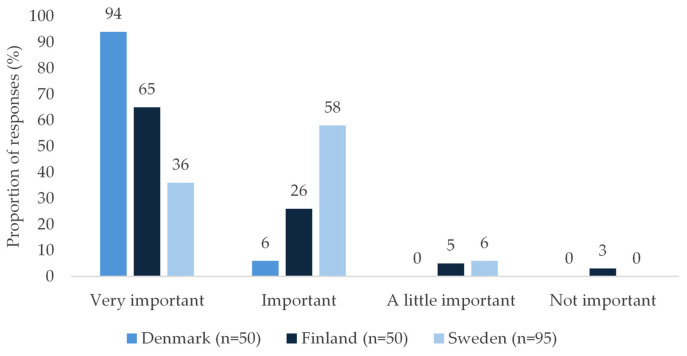
Pharmacy employees’ perception of the importance of AAI shelf life in Denmark, Finland, and Sweden.

All pharmacy employees, except five in Sweden who had no patient contact, participated in the shelf-life perception survey. Pharmacy employees across all participating countries reported that shelf life is an important consideration when recommending AAIs to patients (Figure 2). In Denmark, all respondents considered shelf life important or very important on a 4-point scale (very important, important, a little important, not important). The corresponding proportions were 94% in Finland and 91% in Sweden. When asked whether shelf life represents a general challenge in dispensing AAIs, 96% of Danish employees and 94% of Finnish employees agreed, compared with 46% in Sweden.

Employees estimated that 50.5% of patients in Denmark, 68.5% in Finland, and 48% in Sweden enquire about remaining AAI shelf life during purchase. Most employees had also encountered at least one patient who declined to purchase an AAI because of insufficient remaining shelf life; the proportions were 76% in Denmark, 88% in Finland, and 77% in Sweden.

In Norway, pharmacy staff estimated that approximately 70% of patients ask about expiry before purchasing, and 15% refuse to buy the offered device due to short remaining shelf life.

## 4. Discussion

This study provides the first evidence on the real-world shelf life of AAIs available at Nordic pharmacies, demonstrating that it is substantially shorter than the manufacturing shelf life of 24 months for 300 µg devices and 18 or 19 months for the 150 µg devices [29,30,31,32]. Only 16.8% of AAIs in Denmark, Finland, and Sweden had a remaining shelf life of one year or longer. Consequently, to comply with guidelines, many patients would need to renew their prescriptions twice annually [3,34,35,36,37,38]. A similar pattern was observed in Norway, where only 32.6% of identified devices had a remaining shelf life of one year or longer.

The 150 µg dose was particularly affected, partly due to its inherently shorter shelf life. This disproportionally impacts caregivers of children at risk of anaphylaxis; a group already considered vulnerable due to challenges recognising and responding to anaphylactic emergencies. In Finland, the average remaining shelf life of 4.3 months (median 4.5 months) corresponds to only 24% of the original shelf life, highlighting an especially problematic situation for paediatric patients and their caregivers.

Short remaining shelf life has several practical and clinical consequences. Frequent prescription renewals increase direct and indirect costs for patients and families and introduce logistical barriers that may disproportionally burden both patients and caregivers. These challenges may reduce adherence to the recommendation from EMA to always carry two devices [38].

Limited shelf life also increases the likelihood that patients carry an expired device or have no device available at all. Delayed or absent access to adrenaline is associated with increased healthcare utilisation and higher societal costs, and although expired AAIs still contain some adrenaline, a therapeutically effective dose cannot be guaranteed, and no controlled clinical trials have evaluated their efficacy [17,18,39]. Thus, patients are clearly advised not to use AAIs after expiry, but to replace AAIs before expiry [29,30,31,32].

To our knowledge, this study represents the first systematic analysis of real-world shelf life of AAIs at the pharmacy level in a Nordic setting. Importantly, the shelf life measured in this study reflects availability prior to dispensing; the remaining shelf life at the time the device is handed to the patient is likely even shorter. These findings should therefore be interpreted as best-case estimates of the conditions under which patients acquire AAIs.

Repetition of this study would help confirm whether these findings represent a persistent trend. However, anecdotal evidence suggests that short AAI shelf life has long been a concern among both patients and clinicians. Our findings support this: most pharmacy employees in Denmark and Finland reported that AAI shelf life is a general challenge. The high frequency of patient enquiries about remaining shelf life underscores that expiry dates are highly top-of-mind to patients. Notably, between 76% and 88% of pharmacy employees in Denmark, Finland, and Sweden reported having encountered at least one patient refusing to purchase a potentially life-saving device due to insufficient remaining shelf life—clearly demonstrating that shelf life influences patient behaviour and may undermine adherence to clinical guidelines.

Despite this attention to shelf life, there are no formal requirements obliging pharmacists to routinely inform patients about the remaining shelf life of dispensed medicines. Although licensed healthcare professionals are generally required to perform their duties responsibly and with professional care, this obligation only implicitly suggests that pharmacists should inform patients when medicines have a short remaining shelf life [40,41,42,43]. Whether the near-expiry AAIs identified in the study ultimately reached patients remains uncertain. Pharmacies are required to monitor their inventory and remove expired products from saleable stock, which may also include discarding medicines approaching expiry; however, medicines may still be dispensed as long as they remain within their labelled shelf life. While this practice may raise ethical concerns, particularly for critical medicines such as AAIs, it is not prohibited and could be justified during shortages. In all four Nordic countries, patients are encouraged to return expired medicines, including AAIs, to pharmacies for safe disposal, but these are generally not eligible for refund.

Several limitations should be considered when interpreting these findings. The study included a subset of pharmacies, which may not fully reflect national stock patterns in each country and was not designed to produce a statistically representative national estimate of remaining AAI shelf life. Unobserved differences in local stock management practices, pharmacy size, supply chain dynamics, and surrounding socioeconomic or geographical characteristics may have influenced the observed results. In addition, the data collected did not allow pharmacies to be reliably classified according to factors, such as urban versus rural location or the income level of the surrounding area, precluding stratified analyses along these dimensions. Given the descriptive study objective and the non-probabilistic sampling strategy, inferential statistical testing was not undertaken. Larger-scale studies using representative sampling frames could apply inferential methods to formally compare countries or pharmacy subgroups and assess statistical significance.

With respect to the timing of data collection, data were collected between late July and mid-October 2025. Evidence from Finland, for example, demonstrates pronounced seasonality in prescriptions, with peaks occurring between April and June, corresponding to an increased risk of venom-induced anaphylaxis during the summer months [44]. Given the similar climates in Denmark, Sweden, and Norway, this seasonal pattern is likely applicable to these countries as well. Such variation may result in higher turnover at wholesalers and pharmacies during peak periods, thereby possibly influencing the remaining shelf life of AAIs at the point of dispensing. Extending the data collection period would help clarify the impact of the seasonal prescription fluctuations on observed shelf-life patterns.

Finally, some limitations relate to data sources and measurement. Employee-reported perceptions were based on subjective experience and may therefore be subject to recall bias. In addition, the Norwegian data included information on the AAI batches available at the time of visit, but not complete information on all units in stock. Combining objective stock data with more detailed pharmacy-level information and extended observation periods may help mitigate these limitations in future research.

Despite these limitations, the consistency of findings across all four countries supports the robustness of the main conclusions.

The relatively low at-pharmacy AAI stock observed in Denmark, Finland and Sweden is not unexpected, given the generally frequent and timely deliveries from wholesalers [45,46,47,48]. Wholesaler turnover and manufacturing release timings determine what AAIs are made available to pharmacies and therefore influence the remaining shelf life of products at the point of dispensing. Wholesalers in all four countries are required to comply with European Good Distribution Practices which recommends distribution according to the “first-expiry, first-out” principle [45]. As a result, pharmacies are preferentially supplied with AAIs from the inventory with the shortest remaining shelf life.

The objective of this analysis was not to determine the relative contribution of each the factors determining the shelf life of AAIs at pharmacies available to patients, but to describe the current reality facing patients when attempting to obtain an AAI. Further research could include assessments of manufacturer release timing, wholesaler inventory turnover, and pharmacy stock management practices. Regardless of the mechanisms, products with shorter manufacturing shelf life are inherently more sensitive to delays and inefficiencies in the distribution chain. As such, emergency treatment options with longer shelf life at manufacturing could meaningfully reduce replacement frequency and improve adherence among patients at risk of anaphylaxis.

## 5. Conclusions

This study demonstrates that the real-world shelf life of AAIs available at Nordic pharmacies is consistently and substantially shorter than their manufacturing shelf life. Across all countries and for both doses, the average remaining shelf life at the point of dispensing was below 12 months. As a result, many patients and caregivers receive devices that may require multiple replacements per year, with potential consequences for adherence to clinical guidelines, costs, and practical burden.

For pharmacy managers and policymakers, these findings highlight remaining shelf life as a practical determinant of patient access and adherence, with implications for dispensing practices, replacement frequency, and healthcare system costs. Emergency treatment options with longer manufacturing shelf life may help reduce the risk of non-carriage or reliance on expired AAIs.

## Figures and Tables

**Table 1:** AAIs in stock at pharmacies in Denmark, Finland, and Sweden.

	Denmark	Finland	Sweden	Total
Number of pharmacies	50	50	100	200
Total number of AAIs	128	220	444	792
Number of 150 µg AAIs	46	73	82	201
Number of 300 µg AAIs	82	147	362	591
Pharmacies with 150 µg in stock, %	60%	70%	39%	52%
Pharmacies with 300 µg in stock, %	64%	100%	98%	90%
Average 150 µg AAIs per pharmacy *	1.5	2.0	2.1	1.9
Average 300 µg AAIs per pharmacy *	2.6	2.9	3.7	3.2

* Only includes pharmacies with the relevant dose in stock.

**Table 2:** Remaining shelf life for Denmark, Finland, and Sweden.

	Denmark	Finland	Sweden	Total
Mean duration until expiry, combined doses, months	6.9	8.9	10.7	9.6
Mean duration until expiry, 150 µg, months	6.5	4.3	7.7	6.2
Mean duration until expiry, 300 µg, months	7.2	11.2	11.4	10.8
Total median duration until expiry, months	7.1	9.5	10.7	9.9
Median duration until expiry, 150 µg, months	5.5	4.5	7.8	4.7
Median duration until expiry, 300 µg, months	8.5	9.7	10.7	10.5
Range of total duration until expiry, months	1.1–14.8	1.6–18.0	2.7–21.9	1.1–21.9
Range of remaining shelf life, 150 µg, months	1.1–14.8	1.7–6.9	2.7–21.9	1.1–21.9
Range of remaining shelf life, 300 µg, months	1.2–12.1	1.6–18.0	3.7–18.9	1.2–18.9

## Data Availability

The datasets generated and/or analysed during the current study are proprietary to ALK-Abelló and are not publicly available due to commercial restrictions. Summary results are provided in the manuscript. Anonymised or aggregated extracts that support the findings may be made available on request to the corresponding author.

## Appendix A

**Table A1:** Questions to pharmacy employees of shelf-life perception.

	Question
1.	Do you experience shelf life as a general challenge for adrenaline auto-injectors?
2.	Have you experienced a buyer of adrenaline auto-injector deciding not to purchase the product due to insufficient shelf life?
3.	What percentage of buyers * of adrenaline auto-injectors actively ask about the shelf life of the product they are purchasing? Please give your best estimate.
4.	How important is the shelf life for the recommendation of adrenaline auto-injectors to customers?

* In this table, “buyers” refers to patients or caregivers purchasing AAIs.

**Table A2:** Pharmacy employee role distribution.

	Pharmacist, n	Pharmacy Assistant, n	Other, n
Denmark	17	29	4
Finland	48 *	2	0
Sweden	84	3	13

* In Finland, respondents who identified themselves as ‘proviisori’ and ‘pharmacist’ were grouped.
